# Morphological, Physiological and Photophysiological Responses of Critically Endangered *Acer catalpifolium* to Acid Stress

**DOI:** 10.3390/plants10091958

**Published:** 2021-09-19

**Authors:** Yuyang Zhang, Tao Yu, Wenbao Ma, Buddhi Dayananda, Kenji Iwasaki, Junqing Li

**Affiliations:** 1 The National-Local Joint Engineering Laboratory of High Efficiency and Superior-Quality Cultivation and Fruit Deep Processing Technology on Characteristic Fruit Trees, College of Plant Science, Tarim University, Alear 843300, China; muyu64@sina.com; 2 Beijing Key Laboratory for Forest Resources and Ecosystem Processes, Beijing Forestry University, Beijing 100083, China; yutao123@bjfu.edu.cn; 3 Ecological Restoration and Conservation of Forests and Wetlands Key Laboratory of Sichuan Province, Sichuan Academy of Forestry, Chengdu 610081, China; 4 School of Agriculture and Food Sciences, The University of Queensland, Brisbane, QLD 4072, Australia; b.dayananda@uq.edu.au; 5 Climate Change Cluster (C3), Faculty of Science, University of Technology Sydney, Sydney, NSW 2007, Australia; Kenji.Iwasaki@alumni.uts.edu.au

**Keywords:** acid stress, morphological characteristics, photosynthetic capacity, *Acer catalpifolium*, critically endangered species

## Abstract

Acid rain deposition (AR) has long-lasting implications for the community stability and biodiversity conservation in southwest China. *Acer catalpifolium* is a critically endangered species in the rain zone of Western China where AR occurs frequently. To understand the effects of AR on the morphology and physiology of *A. catalpifolium*, we conducted an acid stress simulation experiment for 1.5 years. The morphological, physiological, and photosynthetic responses of *A. catalpifolium* to the acidity, composition, and deposition pattern of acid stress was observed. The results showed that simulated acid stress can promote the growth of *A. catalpifolium* via the soil application mode. The growth improvement of *A. catalpifolium* under nitric-balanced acid rain via the soil application mode was greater than that of sulfuric-dominated acid rain via the soil application mode. On the contrary, the growth of *A. catalpifolium* was significantly inhibited by acid stress and the inhibition increased with the acidity of acid stress applied via leaf spraying. The inhibitory impacts of nitric-balanced acid rain via the leaf spraying of *A. catalpifolium* were greater than that of sulfur-dominant acid rain via leaf spraying. The observations presented in this work can be utilized for considering potential population restoration plans for *A. catalpifolium*, as well as the forests in southwest China.

## 1. Introduction

Acid rain deposition (AR) has detrimental impacts on the functions of both terrestrial and aquatic ecosystems [1,2]. Sulfur dioxide and nitrogen oxide are major causative agents of acid rain resulting from anthropic emissions, such as the combustion of fossil fuels, agricultural production, and vehicle emissions [3]. Acid rain has been shown to significantly impede plant growth. For example, Lee et al. reported that the total biomass, shoot height, root length, and leaf area were significantly reduced in AR-treated plants at different stages of development [4]. Moreover, Chen et al. observed that acid rain could lead to leaf necrosis [5]. Other works observed various impacts: physiological processes including altering the permeability of plant cells, electrolyte leakage, and the inhibition of transpiration [4,6]; decreasing photosynthetic rates and chlorophyll contents [5,7], affecting leaf nutrient balance [8]; and altering the activities of antioxidant enzymes and the free amino acid composition [5,9].

During the 1980s, the southern regions of China, including the southwestern forests, recorded high levels of AR [10], making China the country with the third-highest level of recorded AR [2,11] with recorded average pH values between 3.5 to 4.8 [12,13]. Acid rain deposition triggered a series of environmental issues in China, including the acidification of soil and water [14,15], plant damage, forest decline [2], and the loss of biodiversity [16]. The Sichuan Basin is one of the most intensive AR regions in China due to high environmental pollution [17]. The extent of the damage caused by acid rain affects up to one third of the whole forestry area in Sichuan with up to 15,000 hm^2^ of severely damaged forest area, or 6% of the total forestry area [16]. Despite being a hotspot of biodiversity, the biodiversity loss of Sichuan due to AR has scarcely been researched. The decrease in species richness with the increase in acid rain is confirmed [18], which may link to the vulnerability of endangered species [19]. The population size of *Liquidambar formosana* and *Schima superba* decreased during the past decade at Dinghushan Biosphere Reserve and serious damage to *L. formosana* caused by AR was found in several southern subtropical forests [7]. A comprehensive understanding of the potential impacts of AR on endangered plants could provide valuable information for plant protection and conservation.

*Acer catalpifolium* is a deciduous broad-leaved tree species native to the southern Chinese region of Sichuan and has been listed in the “Wild Plant with extremely small populations (WPESP) rescue and protection plan” [20]. Young trees are rarely seen in the region due to intensive interspecies competition and understory micro-environments impeding the natural regeneration process of *A. catalpifolium* [21], coupled with a relatively low seed germination rate [22]. Young trees or seedlings are highly sensitive to environmental changes and studies on seedlings of *A. catalpifolium* could help improve our understanding of how they respond to environmental stresses such as AR. As deciduous broad-leaved tree species are sensitive to AR [5], it is very important to evaluate the potential effects of AR on the growth and development of the critically endangered species *A. catalpifolium*.

In this study, we conducted a series of pot experiments in Sichuan province, to analyze the changes in phenotype (plant height, specific leaf area (SLA), and biomass) and photosynthetic capacity (gas exchange indices and chlorophyll content) characteristics under acid stress and to assess the impact of deposition patterns and acid composition. These results will help to reveal the possible mechanism for the effect of acid rain deposition on *A. catalpifolium*, as well as provide practical implications on the protection of endangered plant species against acid stress, and guide conservation efforts of local biodiversity.

## 2. Results

### 2.1. Response of Morphological Characteristics of Acer catalpifolium to Different Forms of Acid Stress

#### 2.1.1. Response of Plant Height to Different Forms of Acid Stress

**Figure 1:**
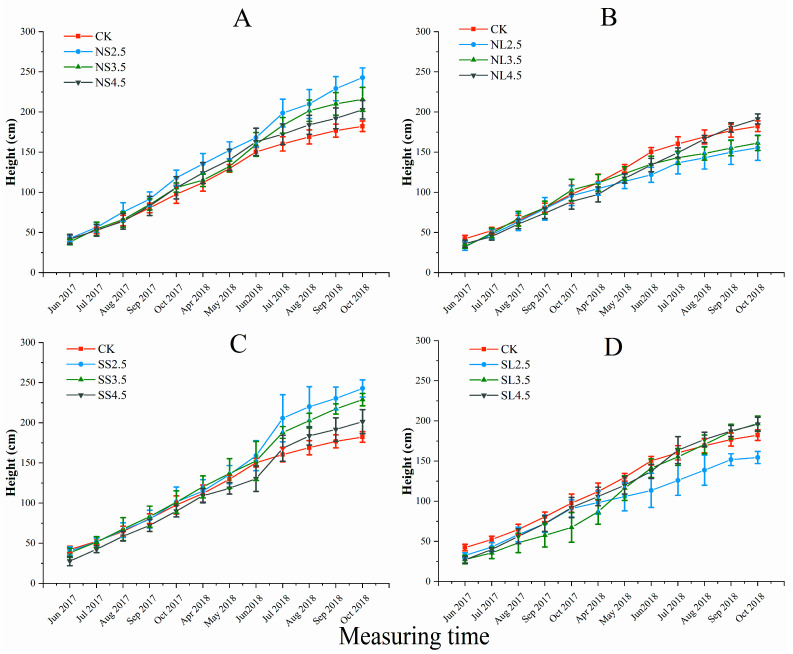
Effect of different forms of acid stress on plant height (cm) of *A. catalpifolium* during the 16-month observation period. The treatments: CK, NS (**A**), NL (**B**), SS (**C**) and SL (**D**) are defined as control, nitric-balanced acid applied to soil, nitric-balanced acid applied to leaves, sulfuric-dominated acid applied to soil and sulfuric-dominated acid applied to leaves, respectively. Values are the average ± standard deviation (n = 10).

The investigated forms of acid stress significantly influenced the plant height in October 2018 (Table S1). The plant height increased as the acidity decreased under both treatments of nitric-balanced acid applied to soil (NS) and sulfuric-dominated acid applied to soil (SS), while the opposite trend was observed in nitric-balanced acid applied to leaf (NL) and sulfuric-dominated acid treatments applied to leaves (SL) (from June 2018 to October 2018) (Figure 1). The plant heights under pH 2.5 and pH 3.5 of NS and SS treatments in October 2018 were significantly higher than those of NL and SL (*p* < 0.05).

#### 2.1.2. Response of Diameter of Ground Stem to Different Forms of Acid Stress

**Figure 2:**
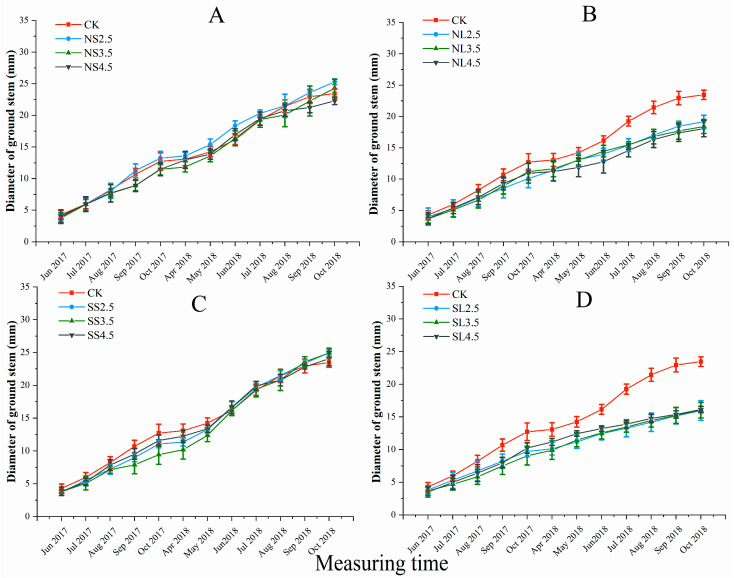
Effect of different forms of acid stress on diameter of ground stem (mm) of *A. catalpifolium* during the 16-month observation period. The treatments: CK, NS (**A**), NL (**B**), SS (**C**) and SL (**D**) are defined as control, nitric-balanced acid applied to soil, nitric-balanced acid applied to leaves, sulfuric-dominated acid applied to soil and sulfuric-dominated acid applied to leaves, respectively. Values are the average ± standard deviation (n = 10).

The maximum ground stem diameter was observed in NS2.5 (25.29 ± 0.48 cm) and SS 2.5 (24.96 ± 0.51 cm) treatment in October 2018 (Figure 2A,C), but no significant difference was observed among the different acidity treatments and control checks (CK). The acid application method and its interactive effects with acid types or acidity had a significant impact on the ground stem diameters of *A. catalpifolium* (Table S1), the leaf spray applications of NL and SL were significantly lower than that of CK (Figure 2B,D), while soil application (NS and SS, respectively) resulted in significantly higher stem diameter than that of leaf spray application (NL and SL, respectively) under the same pH treatment. The ground stem diameters of NS2.5, 3.5, 4.5 and SS2.5, 3.5, 4.5 were 31.89%, 31.93%, 23.38% and 56.29%, 55.63%, 49.28% higher than those of corresponding NL2.5, 3.5, 4.5 and SL2.5, 3.5, 4.5 treatments, respectively, in October 2018.

#### 2.1.3. Response of Crown to Different Forms of Acid Stress

**Figure 3:**
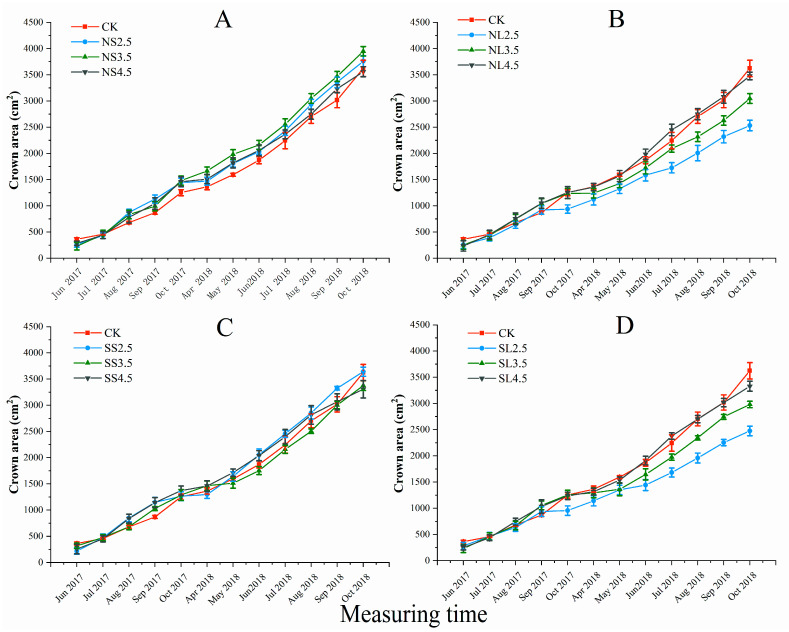
Effect of different forms of acid stress on the crown of *A. catalpifolium* during the 16-month observation period. The treatments: CK, NS (**A**), NL (**B**), SS (**C**) and SL (**D**) are defined as control, nitric-balanced acid applied to soil, nitric-balanced acid applied to leaves, sulfuric-dominated acid applied to soil and sulfuric-dominated acid applied to leaves, respectively. Values are the average ± standard deviation (n = 10).

Based on a three-way analysis of variance, the acid application method and its interactive effects with acid types or acidity had a significant impact on the crown of *A. catalpifolium* in October 2018 (Table S1). The crown width was significantly larger under NS treatments compared to SS (Figure 3A,C). As for leaf spray application, NL2.5 and SL2.5 had significantly smaller crown widths compared to NL3.5, NL4.5, SL3.5, SL4.5, and CK after June 2018 (Figure 3B,D). The crown width of NL2.5 and NL3.5 treatments were significantly lower than that of pH 4.5 treatment and CK.

#### 2.1.4. Leaf Morphological Characteristics

The interactive effects of acid application methods and acidity had a significant impact on the morphological characteristics of *A. catalpifolium* leaves (Table S1). The leaf weight of the NL3.5 and NS3.5 treatments was significantly lower than the rest of the treatments (Table 1). The large leaf area and specific leaf area (SLA) were found in the low acidity of NS treatments in which SLA decreased as the pH increased. In contrast, the SLA of NL treatment increased with the increase in pH, and the NL4.5 and NL2.5 treatments significantly differed from CK. The SLA of SS2.5 treatment was significantly higher than that of SS 3.5 treatment and CK. The SLA of SL2.5 treatment was significantly less than those under SL 4.5 and SL3.5 treatments. The longest leaf length was observed in NS2.5, SS2.5 and SS3.5 treatments, while NS4.5 and SS4.5 treatments resulted in the smallest leaf length. Contrarily, the smallest leaf length in the leaf spray application was observed in both NL2.5 and SL2.5, while the longest leaf length was recorded in CK.

### 2.2. Growth Response of Acer catalpifolium to Different Forms of Acid Stress

Similar to the morphological parameters of leaves, the interactive effects of acid application methods and acidity had a significant impact on the biomass parameters of *A. catalpifolium* (Table S1). The soil application, root biomass, leaf biomass, lateral branch biomass, stem biomass, and total biomass of *A. catalpifolium* decreased with the increasing pH, the opposite trend observed in the leaf spray application treatment (Table 2). The root biomass, stem biomass, and total biomass in NS2.5 and SS2.5 treatments were significantly higher compared to other treatments including CK. Leaf biomass of NS2.5 and SL4.5 treatment were significantly larger than other treatments, including CK. The lateral branch biomass of SL4.5 treatments was significantly higher than that of CK, but the lateral branch biomass of SS4.5, NS4.5, NL2.5, and NL3.5 treatments were significantly lower compared to CK.

**Figure 4:**
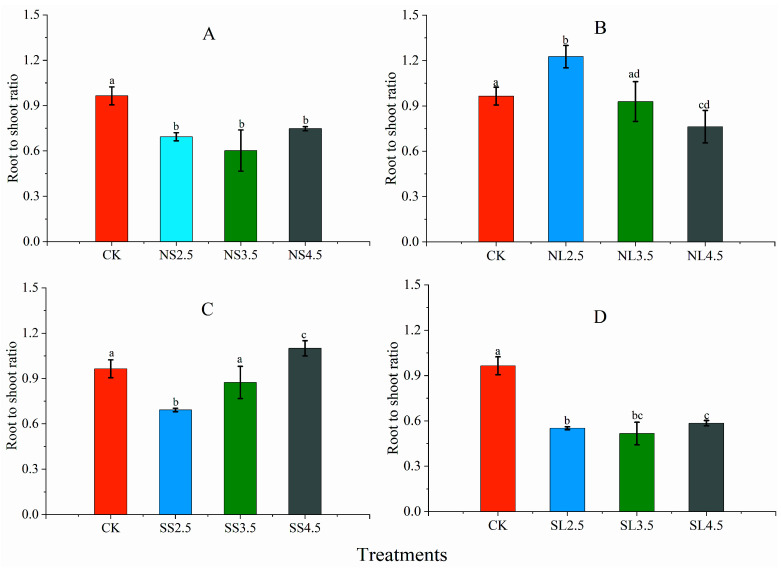
Effect of different forms of acid stress on root-to-shoot ratio (RSR) of *A. catalpifolium*. The treatments: CK, NS (**A**), NL (**B**), SS (**C**) and SL (**D**) are defined as control, nitric-balanced acid applied to soil, nitric-balanced acid applied to leaves, sulfuric-dominated acid applied to soil and sulfuric-dominated acid applied to leaves, respectively. Values are the average ± standard deviation (n = 10). Different letters above the error bars in each faceted plots indicated a significant difference (*p* < 0.05) among different acidity within the same acid treatments.

The root-to-shoot ratios (RSR) of NS2.5, NS3.5, NS4.5, SL2.5, SL3.5, and SL4.5 were 28.01%, 37.50%, 22.49%, 42.79%, 46.44%, and 39.31% lower compared to CK, respectively (Figure 4A,D), while the RSR of NL treatments decreased with the increasing of treatment pH, the RSR of NL4.5 was significantly lower than that of CK, and NL2.5 showed the opposite trend. The RSR of SS treatments increased with increasing pH, the RSR of SS2.5 and SS4.5 significantly differed from CK.

### 2.3. Effects of Different Forms of Acid Stress on Photosynthetic Characteristics of Acer catalpifolium

Acid types and its interactive effects with acid application methods and acidity had significant influence on photosynthetic characteristics of *A. catalpifolium* (Table S1). The observed net photosynthetic rate (*P*n) in soil application treatments increased as pH decreased as the highest *P*n were observed in NS2.5 and SS2.5 (2.87 ± 0.040 and 2.43 ± 0.267 μmol m^−2^ s^−1^, respectively) (Table 3). For the leaf spray application, NL and SL treatments were all significantly lower than that of CK (1.75 ± 0.019 μmol m^−2^ s^−1^), NL2.5, NL3.5, NL4.5, SL2.5, SL3.5, SL4.5 were 17.71%, 10.86%, 13.14%, 29.08%, 27.94%, and 17.71% lower than CK, respectively, while soil application treatments showed contrary results (Table 3). The transpiration rates (*T*r) of NL treatments were higher than that of CK, where the lowest *T*r observed in the SL2.5 treatment was 0.117 ± 0.001 mmol H_2_O^−2^ s^−1^. The maximum stomatal conductance (*g*s) that appeared in the treatment of NS2.5 was 0.014 mol CO_2_ m^−2^ s^−1^; as for leaf spray application, the gs in NS treatments decreased with the increase in treatment pH, but SS showed the opposite results. Simulated acid stress treatments significantly influenced water use efficiency (WUE); the lowest WUE occurred in SL3.5 treatment (9.43 ± 0.30), which was significantly different from CK. The treatments, SL2.5, SL4.5, and all of SS and NS WUE, decreased with the increasing pH. The intercellular CO_2_ concentrations (*C*i) in the NL and SL treatments were significantly greater than that of CK; however, the *C*i in NS and SS treatments were significantly lower than CK, except for SS2.5 (178.05 ± 13.53 μmol^−1^ mol). The stomatal limitations (*L*s) of NL differed in various acidity treatments and were significantly lower than that of CK. The *L*s in SL treatments increased with the treatment pH, in which SL treatments were significantly smaller than CK. The *L*s changes in NS and SS treatments were significantly higher than that of CK (except for NS4.5, 0.557 ± 0.014).

### 2.4. Effects of Different Forms of Acid Stress on Chlorophyll Content of Acer catalpifolium

**Figure 5:**
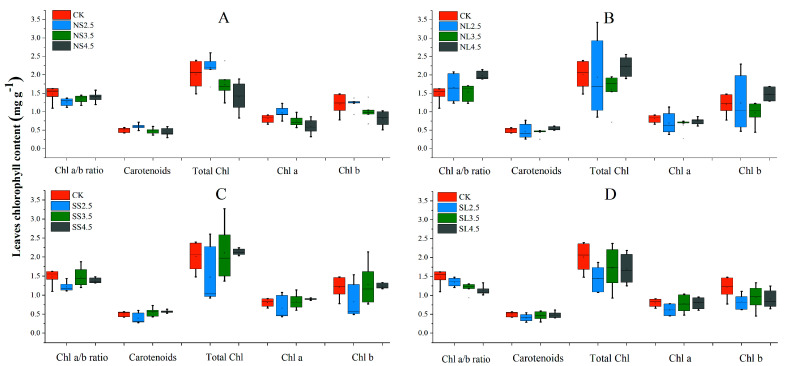
Effect of different forms of acid stress on different species’ chlorophyll content of *Acer catalpifolium* leaves. The treatments: CK, CK, NS (**A**), NL (**B**), SS (**C**) and SL (**D**) indicated are defined as control, nitric-balanced acid applied to soil, nitric-balanced acid applied to leaves, sulfuric-dominated acid applied to soil and sulfuric-dominated acid applied to leaves, respectively. The black dot in each of the boxes represents mean values and bars and stand for maximum or minimum value of each treatment (n = 10).

The interactive effects of acid application methods, acid types and acidity had a significant impact on the chlorophyll content of *A. catalpifolium* (Table S1). The total chlorophyll content, carotenoids, chlorophyll *a* (Chl *a*), and chlorophyll *b* (Chl *b*) under NS treatments decreased with the increasing pH. The contents of carotenoids, total chlorophyll, Chl *a,* and Chl *b* in NS2.5 were significantly higher than those in NS3.5 and NS4.5, with values of 20.51%, 27.18%, 20.89%, 35.88%, 24.30%, 38.72%, 18.17% and 33.61%, respectively. While SS treatments showed the opposite trends; carotenoids, total chlorophyll content (Figure 5B), Chl *a,* and Chl *b* under NL with pH 2.5 treatment were higher compared to other acidity treatments of NL, while NL with pH 3.5 treatment showed similar results (Figure 5C). The Chl *a*/*b* ratio of the CK was 1.48 ± 0.20 mg g^−1^; the Chl *a*/*b* ratio under NS treatments significantly increased with treatment pH. Yet, SL treatments observed the opposite results, where the chlorophyll *a*/*b* (Chl *a*/*b*) of NS2.5, SS2.5, SL3.5 and SL4.5 were 14.51%, 17.43%, 18.95% and 24.05%, respectively, significantly lower than that of CK; the Chl *a*/*b* of NL4.5 was 34.12%, significantly higher than that of CK (Figure 5C,D).

The foliar chlorophyll parameters (including carotenoids, total Chl, Chl *a,* and Chl *b*) were positively related to *P*n, *g*s, *T*r, and WUE under different acid treatments (Figure S1). Ls had a negative relation to foliar chlorophyll parameters. The contents of foliar total Chl, Chl *a,* and Chl *b* of *A. catalpifolium* were positively related to carotenoids despite of different acid treatments (Figure S1).

## 3. Discussion

The growth parameters of *A. catalpifolium* were significantly affected during the one-year application of different acid treatments. Previous studies established that nutrient (primarily nitrogen and sulfur) deposition increased along with the rapid growth of the acidity of the acid treatments and facilitated plant growth [23,24]. It is well interpreted that the acidity of acid treatments and its interactive effects with acid application methods showed great positive impacts on plant height, ground stems, crown breadth and the biomass of *A. catalpifolium*, especially when soil with low acidity treatments was applied (such as NS2.5 and SS2.5). In this study, acid application methods simulated two influence pathways of acid rain on plant growth which included the direct effects on plant body and indirect effects from soil-mediated [23]. From the aspect of indirect effects, the biomass of *A. catalpifolium* under soil-applied acid treatments increased with the increasing acidity, which had similar change patterns with *Schima superba* and *Elaeocarpus glabripetalus* [3,24]. Furthermore, inputted N and S from acid rain may have compensated plant development. Additionally, exogenous H^+^ could accelerate nutrient mineralization and extend the soil’s available nutrients pool which had benefits for plant growth [7,25,26]. High net photosynthetic rate in soil application treatments further explained better performance in growth and biomass accumulation [24]. However, the biomass improvement only appeared when treated with a low pH acid rain (the total biomasses of SL4.5 and NL4.5 were 316.30 ± 2.08 g and 309.10 ± 14.88 g), an observation which was reported in previous studies [3,24].

On the contrary, leaf spray treatments had a significantly negative impact on the plant growth; the plant height of *A. catalpifolium* under NL2.5, NL3.5 and SL2.5 was 14.71%, 11.43% and 15.27% lower than that of CK. The plant biomass accumulation also showed a similar trend consistent with previous results [27,28]. Direct acid spraying may damage the key components of the plant [3], resulting in the destruction of physiological functions (such as low *P*n and chlorophyll content) and then slow growth, which was consistent with previous findings [29,30,31].

Leaves were one of the most susceptible components to environmental stress in this study; acid addition significantly changed foliar morphological features (Table S1). Lower leaf length and width was observed in NL2.5 compared to CK (Table 2). This was similar to Percy’s study on *Acer rubrum* which demonstrated that the addition of acid rain with pH 2.5 significantly reduced leaf length, but no significant difference was observed when treating with a high pH acid rain [32]. Previous studies also found significant changes in the foliar internal structure. Sant’Anna-Santos et al. found that acid rain changed epidermal cells of the leaf, resulting in the erosion of the cuticle, and the altering of leaf permeability [33]. Therefore, future study should pay more attention to the changes of the foliar anatomic structure of *A. catalpifolium* and its relation to foliar morphological and physiological parameters.

Photosynthesis is the basic photochemical process for plant survival and development [34]. The interactive effects of acid application method, acid types and acidity had a significant impact on the foliar gas exchange indices (Table S1), in which *P*n under a leaf spray application was significantly lower than that of CK (Table 3). The decreased *P*n further declined with decreasing acidity of acid rain, which was widely reported previously [35,36]. This decline may have been linked to H^+^ accumulation in the leaves causing uncoupled electron transport and the insufficient accumulation of ATP and NADPH [37]. Another possible reason may be due to essential elements (such as magnesium) leaching from the leaves interfering with the biosynthesis of chlorophyll, resulting in low Chl content [38]. Chlorophyll content generally had a strong positive correlation to *P*n (Figure S1). The Chl content in soil application treatments increased with the enhancing acidity treatment, agreeing with results from Huang et al. [39] and Liu et al. [24], which may be associated with the exogenous N input promoting the biosynthesis of Chl. Many researchers supported the idea that environmental stress led to the decrease in *g*s of plants, restricting the introduction of CO_2_ and H_2_O into cells, preventing the transfer of the photosynthetic electron chain and the reduction in chlorophyll content, and hence causing a decline in the photosynthetic rate [3,5,40]. Feng et al. suggested that there was a significantly positive correlation between the net photosynthetic rate and *g*s in subtropical evergreen broad-leaved trees [41], consistent with our findings (Figure S1). However, Momen et al. showed that changes in the photosynthetic rate and *g*s of the leaves subjected to AR were not consistent [42]. Therefore, the factors affecting the photosynthetic rate were complex and varied, and unlikely caused by the *L*s alone. The influences of AR on plant photosynthesis were separated into stomatal factors and non-stomatal factors [41,42], *C*i and *L*s, frequently used for judging limitation factors [41]. When the photosynthetic rate and stomatal closure were consistent, stomatal factors were considered; if the photosynthetic capacity of mesophyll cells was significantly reduced, their ability to use CO_2_ was reduced, thus increasing *C*i and decreasing *L*s, which was considered a typical non-stomatal restriction [41]. In this study, inconsistent changes among *P*n, *C*i, *g*s, and *L*s were observed in the leaf spray application treatments. This can be hypothesized as the deceased *P*n was caused by the non-stomatal restriction, photosynthetic pigments and related enzymes activity had a major contribution to the *P*n changes. Du et al. collected all the available data regarding the impact of simulated acid rain on Chl content, which suggested that the chlorophyll content decreased by 6.71% with increasing pH [2]. The acid stress also damaged chloroplast structure, inhibited the expression of six chloroplast ATP synthase subunits, decreased chloroplast ATP synthase activity, and reduced photosynthesis and plant growth [38]. In this study, each increase or decrease in the pH value of acid rain had different effects on the total chlorophyll content.

The natural habitats of *A. catalpifolium* generally received low pH precipitation [43]. The potential damage or inhibition of growth or physiological functions of *A. catalpifolium* caused by persistent spraying acid to foliar, were observed in the current simulation study. Field evaluation should be conducted specially for in situ conservation programs, and provide suggestions based on plant performance. On the other hand, the fertilization effects of acid rain (the addition of multiple nutrients such as nitrogen and sulfur) should not be neglected.

## 4. Materials and Methods

### 4.1. Experimental Materials

On 10 December 2016, the seeds of *A. catalpifolium* were collected from Dujiangyan, Sichuan province (30°57′22.85″ N; 103°32′37.8″ E; 814 m (Elevation: EV)) and stored at 4 °C (HerryTech LHP300) while preparing for germination. After removing the fruit wings, the seeds were placed in a petri dish and 10 mL of deionized water was added for germination. The petri dish was then stored in an incubator (Haier BCD625WDG) at a constant temperature of 15 °C, air humidity of 60%, and exposed to sunlight for 14 h, on 15 January 2017. After the seeds had germinated, they were transferred to Sichuan Forestry Science Research Institute, Tangchang Town, Hengshan Village Base. Seedbed (with a thickness of 0.3 m, width of 1.0 m, and length of 20.0 m) was used for growing germinated seeds which were filled with soil (classified as Burozem, soil pH of 6.8, soil organic matter of 2.52%, total nitrogen of 0.14%, total phosphorus of 0.24%, total potassium of 0.47%, available phosphorus of 27.21 mg kg^−1^, and available potassium of 134.68 mg kg^−1^) and mixed with forest litter (mainly wood chips) at a ratio of 1:1 (*v*/*v*). The germinated seeds were planted in the surface layer 15 cm apart, watered, and then covered with plastic film to increase their survival rate. There were more than 500 viable and well-grown seedlings. In April 2017, seedlings with similar phenotypes were selected and transplanted to plastic pots (with an inner diameter of 35 cm and a height of 45 cm, 1 plant per pot). All pots were filled with the same substance formula as the seedbed. After observing the growth for 30 days, some pots were removed to maintain a uniform growth pattern across all pots. The average height and base diameter of *A. catalpifolium* seedlings were 34.85 ± 0.58 cm and 3.36 ± 0.25 mm.

### 4.2. Experimental Design

The acid stress started on 1 May 2017 and ended on 31 October 2018 (except December 2017 to February 2018, during the winter dormant period). An acidity range of pH 2.5, 3.5, and 4.5 was used, which was based on the lowest pH (pH 2.5) observed in precipitation recorded in Sichuan since 2000 [10] and the average pH (pH 4.5) of precipitation recorded in Sichuan from 2006–2013 [44]; an intermediate value of pH 3.5 was also considered due to various environmental changes. According to the changes of SO_4_^2-^:NO_3_^−^ ratio in precipitation of this region, pure H_2_SO_4,_ and HNO_3_ were used to formulate two types of solution (dissolved in deionized water), included in the molar ratio of 7.5:1 (sulfur dominant acid rain, SD) which reflected the intermediate value of SO_4_^2-^:NO_3_^−^ (7.5:1) in Sichuan in the past years [10]. The ratio of 5:4.1 (nitric balanced acid rain, NB) was related to the previous sulfur emission mitigation policy (100 kg ha^−1^ sulfur deposition in this area) [45] and the increased importance of N deposition (82.41 kg ha^−1^) [46].

The experimental plants were placed in open shelter to prevent rain entering the plants and soil, and to ensure the microclimate (such as temperature and moisture) under shelter was kept the same as the surrounding area. The experiment conducted in this study was 2 (acid application method) × 2 (acid compositions) × 3 (acid concentration) factors with a blank control check (CK). The control check was grown with activated deionized water without acid treatment. The SD treatments were divided into two groups: group 1 had acid solutions (pH 2.5, 3.5, and 4.5) applied to the soil, designated SS2.5, SS3.5, and SS4.5, respectively; group 2 had acid solutions (pH 2.5, 3.5 and 4.5) sprayed onto the leaves, designated SL2.5, SL3.5 and SL4.5, respectively. The NB treatments were grouped in the same design as SD, designated NS2.5, NS3.5, NS4.5, NL2.5, NL3.5, NL4.5, respectively. The first letters, S and N, stand for SD and NB; the second letters, S and L, stand for soil application mode and leaf spray application, and the numbers 2.5, 3.5, and 4.5 stand for the corresponding pH values. All treatment groups were applied with 300 mL acid solution every 5 days.

The soil layer was covered with a plastic film before spraying the acid solution to leaves in case the acid solution was introduced into the soil, the film was subsequently removed, and 300 mL of deionized water was added to the soil. The control group was simultaneously irrigated with 300 mL of deionized water on the foliage and soil. This study was therefore conducted with a total of 12 treatments and CK, with each treatment represented by an average of 10 seedlings, for a total of 13 treatments with 10 biological replicates.

To avoid variations caused by the position of the plants, the positions of the potted seedlings were changed every 30 days to ensure the homogeneity of each seedling. During the treatment of *A. catalpifolium*, we masked it with a simple plastic greenhouse to prevent natural precipitation from affecting the experimental treatment.

### 4.3. Growth Parameters

During the experiment, monthly measurements were carried out on the height, ground diameter, crown width, and the number of leaves of *A. catalpifolium* young trees. The measurements were collected from June 2017 to October 2017 and April 2018 to October 2018 (morphological characteristics were not measured from November 2017 to March 2018 because of low temperatures which caused slow plant growth). The ground diameter was measured using digital calipers and the plant height was measured with a tape measure. The crown measurement first measured the length of the crown (A) at the maximum extension of each seedling and measured the width of the crown (B) in the vertical direction of the plane. The calculation formula of the crown of *A. catalpifolium* was: Crown = A × B.

In October 2018, two fully expanded leaves of each *A. catalpifolium* were selected to measure the length and width. In October 2018, the same leaves were taken from each plant to measure leaf area and specific leaf area (SLA) using a leaf area utilization scanner (CanonScan Lide 120) and were analyzed using an image analysis software (Image J). The leaves were subsequently oven-dried at 80 °C for 72 h. The whole *A. catalpifolium* young trees were harvested in November 2018, each tree was divided into four parts: root, stem, lateral branches, and leaves; each part was oven-dried at 80 °C, and subsequently weighed. The total stem biomass (W_total_) (1) and root–shoot ratio (RSR) (2) were calculated as follows:

W_total_ = W_stem_ + W_lb_, (1)

RSR = W_root_/(W_stem_ + W_leaf_), (2)

where: W_total_, W_stem_ and W_lb_ represent total stem biomass, stem biomass, lateral branches biomass, respectively; W_root_ and W_leaf_ represent roots and leaf biomass, respectively.

### 4.4. Photosynthetic Characteristics

In September 2018, LI-6400 XT portable photosynthetic system (LI-COR Inc., LI-COR Biosciences, 4647 Superior Street, P.O. Box 4425, Lincoln, NE 68504-0425, United States) with 6400-02b LED red/blue source leaf chamber was adopted to measure the gas exchange index including *P*n, *C*i, *g*s, and *T*r on a sunny morning (9:00–11:00). The conditions of the measuring chamber were set as follows: 500 µmol s^−1^ airflow, 400 µmol CO_2_ mol^−1^ CO_2_ concentration, 26–28 °C leaf temperature, and 800 µmol m^−2^ s^−1^ photosynthetic photon flux densities. The *L*s (3), and WUE (4), were calculated according to the following formulas [47]:

*L_s_* = 1 − *C_i_*/*C_a_*, (3)

WUE = *P_n_*/*T_r_*, (4)

where:$\;L_{S}$, $C_{i}$, $C_{a}$, WUE, $P_{n}$ and $T_{r}$ represent stomatal limit, intercellular CO_2_ concentration, atmospheric CO_2_ concentration, water use efficiency, net photosynthetic rate, and transpiration rate, respectively.

In October 2018, the chlorophyll contents of different treatment leaves were collected. The extraction method used was detailed in Arnon et al. [48] and was modified as detailed in [49]. Fresh leaves were weighed (0.05 g), and 10 mL of acetone:ethanol 1:1 (*v*/*v*) at 20 °C was added. The ultraviolet-visible light spectrophotometer (Shimadzu UV-2600) measured 663 nm ($A_{663}$), 645 nm ($A_{645}$), and 470 nm ($A_{470}$) wavelengths, respectively. Calculate Chl *a* (6), Chl *b* (7), ${\mathrm{TO}}_{chl}$ (8) and Carotenoid (9) by referring to the following equations.

Chlorophyll content was calculated using the following equation detailed in Li. [50]:

Chl *a* (mg g^−1^) = (12.21 × *A*_663_ − 2.81 × *A*_645_) × *V_T_* × *N/*(1000 × *W*), (5)

Chl *b* (mg g^−1^) = (30.31 × *A*_645_ − 5.03 × *A*_663_) × *V_T_* × *N/*(1000 × *W*), (6)

TO *_Chl_* (mg g^−1^) = (7.18 × *A*_663_ + 27.5 × *A*_645_) × *V_T_* × *N/*(1000 × *W*) (7)

Carotenoid (mg g^−1^) = (1000 × *A*_470_ − 3.27 × *Chl a* − 104 × *Chl b*) × *V_T_* × *N/*(1000 × *W* × 229), (8)

where: In each of these formulas, $V_{T}$ is the volume of extract, W is the fresh weight of the sample (0.05 g), and N is dilution factor, respectively.

### 4.5. Data Analysis

The difference in morphological and physiological characteristics of *A. catalpifolium* in different acidic treatments were analyzed by using a one-way analysis of variance (ANOVA) followed by LSD test for post hoc multiple comparisons at a significance level of 0.05. Additionally, three-way analysis of variance was conducted to assess individual or interactive effects of the acid application method (AAM), acid types (AT) and acidity of acid (AA). Pearson correlation analysis was performed to revealed relationship among different foliar morphological and physiological indices under different acidic treatments. The data were statistically analyzed using SPSS 21 and R 4.0.3. The figures were created using OriginPro2016 and corrplot package of R 4.0.3.

## 5. Conclusions

In this study, acid treatments (different composition and concentration) were continuously added to the endangered species *Acer catalpifolium* by using different application methods for 1.5 years to simulate acid rain. The changes of morphological and physiological features indicated that the soil addition of high concentration acid significantly increased plant growth, biomass accumulation and photosynthesis ability of *A. catalpifolium*. However, the high concentration of acid directly applied onto the leaves interfered with plant development and physiological functions. The results had implications for improving the protection of *A. catalpifolium* by selecting a suitable habitat and adjusting the supply of soil nutrients.

## Figures and Tables

**Table 1:** Effect of different forms of acid stress on leaf weight and specific leaf area (SLA) of *A. catalpifolium.* The treatments: CK, NS, NL, SS and SL are defined as control, nitric-balanced acid applied to soil, nitric-balanced acid applied to leaves, sulfuric-dominated acid applied to soil and sulfuric-dominated acid applied to leaves, respectively. Values are the average ± standard deviation (n = 10). Different superscript letters in each column indicated a significant difference among different acid treatments (*p* < 0.05).

Treatments		Leaf Weight (g)	Leaf Area (cm^2^)	SLA (cm^2^ g^−1^)	Leaf Length (cm)	Leaf Width (cm)
CK		1.25 ± 0.23 ^a^	85.72 ± 12.19 ^b^	69.19 ± 4.99 ^c^	14.50 ± 1.38 ^b^	13.88 ± 1.11 ^ab^
NS	2.5	1.10 ± 0.36 ^ab^	87.66 ± 24.61 ^b^	81.80 ± 11.36 ^b^	16.28 ± 2.67 ^a^	14.58 ± 0.57 ^a^
	3.5	0.93 ± 0.22 ^b^	68.83 ± 3.01 ^d^	76.40 ± 13.92 ^bc^	15.20 ± 1.23 ^ab^	13.23 ± 2.00 ^ab^
	4.5	1.33 ± 0.27 ^a^	69.62 ± 15.55 ^bd^	52.18 ± 4.29 ^d^	13.50 ± 1.22 ^b^	12.00 ± 1.09 ^b^
SS	2.5	1.03 ± 0.30 ^a^	53.32 ± 11.44 ^d^	52.85 ± 9.10 ^d^	16.85 ± 1.35 ^a^	14.28 ± 1.28 ^a^
	3.5	1.12 ± 0.31 ^a^	72.67 ± 8.76 ^bc^	67.74 ± 11.75 ^c^	16.65 ± 1.17 ^a^	13.63 ± 0.86 ^a^
	4.5	1.22 ± 0.19 ^a^	73.15 ± 7.59 ^bc^	60.54 ± 4.02 ^c^	15.92 ± 2.59 ^ab^	11.43 ± 1.77 ^b^
NL	2.5	1.20 ± 0.23 ^a^	62.51 ± 9.60 ^bcd^	52.38 ± 2.80 ^d^	12.00 ± 0.82 ^c^	11.71 ± 1.14 ^b^
	3.5	0.85 ± 0.29 ^b^	62.21 ± 17.86 ^bcd^	75.78 ± 14.49 ^c^	12.30 ± 2.21 ^c^	12.02 ± 0.70 ^b^
	4.5	1.20 ± 0.33 ^a^	122.54 ± 27.07 ^a^	104.80 ± 16.62 ^a^	13.87 ± 2.09 ^bc^	13.53 ± 1.15 ^a^
SL	2.5	1.30 ± 0.11 ^a^	77.14 ± 18.33 ^bc^	58.70 ± 10.20 ^d^	12.37 ± 0.72 ^c^	11.80 ± 1.88 ^b^
	3.5	1.22 ± 0.23 ^a^	89.36 ± 15.80 ^b^	73.80 ± 4.69 ^c^	14.40 ± 1.38 ^b^	12.78 ± 1.57 ^b^
	4.5	1.03 ± 0.33 ^a^	73.44 ± 18.11 ^bc^	73.12 ± 8.52 ^c^	16.00 ± 2.28 ^a^	13.62 ± 1.65 ^ab^

**Table 2:** Effects of different forms of acid stress on different parts of biomass and total biomass of *A. catalpifolium.* The treatments: CK, NS, NL, SS and SL are defined as control, nitric-balanced acid applied to soil, nitric-balanced acid applied to leaves, sulfuric-dominated acid applied to soil and sulfuric-dominated acid applied to leaves, respectively. Values are the average ± standard deviation (n = 10). Different superscript letters in each column indicated a significant difference among different acid treatments (*p* < 0.05).

		Root Biomass (g)	Leaf Biomass (g)	Lateral Branch Biomass (g)	Stem Biomass (g)	Total Biomass (g)
CK		92.50 ± 7.78 ^e^	23.05 ± 1.59 ^d^	13.55 ± 1.04 ^e^	85.55 ± 6.52 ^e^	214.65 ± 0.71 ^e^
NS	2.5	180.20 ± 12.93 ^a^	43.90 ± 5.92 ^a^	27.80 ± 2.74 ^c^	187.45 ± 5.42 ^a^	439.35 ± 21.53 ^a^
	3.5	110.70 ± 18.62 ^d^	30.50 ± 2.63 ^b^	15.70 ± 1.31 ^e^	139.45 ± 9.69 ^c^	296.35 ± 7.61 ^c^
	4.5	74.40 ± 15.23 ^f^	28.85 ± 2.03 ^b^	12.35 ± 2.46 ^f^	58.00 ± 18.07 ^hi^	173.60 ± 33.74 ^f^
SS	2.5	139.80 ± 6.02 ^c^	31.30 ± 4.05 ^b^	19.15 ± 0.71 ^d^	151.30 ± 1.97 ^b^	341.55 ± 11.33 ^b^
	3.5	120.00 ± 6.57 ^d^	23.65 ± 1.70 ^d^	14.20 ± 3.61 ^e^	100.35 ± 4.00 ^f^	258.20 ± 2.74 ^d^
	4.5	94.15 ± 3.12 ^e^	9.50 ± 2.74 ^e^	5.20 ± 0.66 ^g^	70.90 ± 2.30 ^g^	179.75 ± 2.03 ^f^
NL	2.5	66.40 ± 8.76 ^f^	6.40 ± 2.30 ^f^	6.75 ± 0.71 ^g^	40.80 ± 6.90 ^i^	120.35 ± 12.65 ^g^
	3.5	87.20 ± 7.12 ^e^	11.20 ± 0.77 ^e^	11.65 ± 1.26 ^f^	71.70 ± 6.24 ^g^	181.75 ± 1.37 ^f^
	4.5	151.85 ± 11.93 ^b^	35.80 ± 4.05 ^d^	30.75 ± 1.37 ^b^	90.70 ± 5.92 ^e^	309.10 ± 14.88 ^b^
SL	2.5	58.15 ± 8.38 ^f^	26.15 ± 1.04 ^c^	13.45 ± 1.26 ^e^	66.00 ± 16.87 ^g^	163.75 ± 25.47 ^f^
	3.5	88.70 ± 6.24 ^e^	38.70 ± 1.10 ^a^	33.50 ± 2.74 ^ab^	101.00 ± 16.76 ^de^	261.90 ± 6.68 ^d^
	4.5	116.80 ± 2.96 ^d^	40.65 ± 0.82 ^a^	39.75 ± 4.44 ^a^	119.10 ± 6.13 ^d^	316.30 ± 2.08 ^b^

**Table 3:** Effect of different forms of acid stress on gas exchange index of *A. catalpifolium.* The treatments: CK, NS, NL, SS and SL are defined as control, nitric-balanced acid applied to soil, nitric-balanced acid applied to leaves, sulfuric-dominated acid applied to soil and sulfuric-dominated acid applied to leaves, respectively. Values are the average ± standard deviation (n = 10). Different superscript letters in each column indicate a significant difference among different acid treatments (*p* < 0.05).

Treatments		*P*n (μmol m^−2^ s^−1^)	*g*s (mol CO_2_ m^−2^ s^−1^)	*C*i (μmol^−1^ mol)	*T*r (mmol H_2_O^−2^ s^−1^)	*L*s	WUE
CK		1.75 ± 0.019 ^f^	0.009 ^f^	170.80 ± 4.41 ^d^	0.144 ± 0.001 ^d^	0.571 ± 0.011 ^c^	12.18 ± 0.17 ^f^
NS	2.5	2.87 ± 0.040 ^a^	0.014 ^a^	154.27 ± 4.17 ^e^	0.123 ± 0.001 ^g^	0.663 ± 0.011 ^b^	17.01 ± 0.246 ^b^
	3.5	2.30 ± 0.085 ^c^	0.011 ^d^	162.21 ± 12.26 ^e^	0.143 ± 0.001 ^c^	0.592 ± 0.031 ^c^	16.12 ± 0.64 ^c^
	4.5	2.16 ± 0.047 ^d^	0.011 ^d^	156.13 ± 5.54 ^e^	0.162 ± 0.001 ^a^	0.557 ± 0.014 ^c^	13.39 ± 0.26 ^e^
SS	2.5	2.43 ± 0.267 ^b^	0.008 ^g^	178.05 ± 13.53 ^d^	0.132 ± 0.002 ^e^	0.719 ± 0.029 ^a^	18.44 ± 1.12 ^a^
	3.5	2.07 ± 0.057 ^e^	0.009 ^f^	124.40 ± 8.62 ^f^	0.126 ± 0.001 ^f^	0.686 ± 0.022 ^b^	16.39 ± 0.42 ^c^
	4.5	2.10 ± 0.041 ^e^	0.009 ^f^	128.03 ± 7.58 ^f^	0.144 ± 0.001 ^d^	0.677 ± 0.019 ^b^	14.64 ± 0.35 ^d^
NL	2.5	1.44 ± 0.151 ^h^	0.012 ^c^	300.13 ± 4.15 ^a^	0.149 ± 0.005 ^c^	0.503 ± 0.017 ^d^	9.74 ± 1.04 ^g^
	3.5	1.56 ± 0.032 ^g^	0.013 ^b^	271.02 ± 3.71 ^b^	0.157 ± 0.001 ^b^	0.570 ± 0.009 ^c^	16.28 ± 0.20 ^c^
	4.5	1.52 ± 0.035 ^g^	0.012 ^c^	260.76 ± 5.21 ^b^	0.145 ± 0.001 ^d^	0.595 ± 0.013 ^c^	17.41 ± 0.23 ^b^
SL	2.5	1.241 ± 0.035 ^i^	0.009 ^f^	268.13 ± 5.38 ^b^	0.117 ± 0.001 ^h^	0.326 ± 0.013 ^f^	10.62 ± 0.26 ^g^
	3.5	1.261 ± 0.034 ^i^	0.010 ^e^	282.72 ± 7.30 ^b^	0.134 ± 0.001 ^e^	0.290 ± 0.018 ^g^	9.43 ± 0.30 ^g^
	4.5	1.44 ± 0.036 ^h^	0.009 ^f^	239.70 ± 5.22 ^c^	0.135 ± 0.002 ^e^	0.398 ± 0.013 ^e^	10.69 ± 0.20 ^g^

Where: *P*n, *g*s, *C*i, *T*r, *L*s, WUE represent net photosynthetic rate, stomatal conductance, intercellular CO_2_ concentration, transpiration rate, stomatal limitation, and water use efficiency.

## Data Availability

Not applicable.

## Supplementary Materials

The following are available online at https://www.mdpi.com/article/10.3390/plants10091958/s1, Figure S1: Correlation analysis of different foliar morphological and physiological indices of *Acer catalpifolium* under different acidic treatments., Table S1: Three-way analysis of variance for the effects of acid treatments on growth, leaf morphological, biomass, gas eachange and chlorophyll parameters of *A. catalpifolium*.
