# Supplementary material for: Morphological, Physiological and Photophysiological Responses of Critically Endangered Acer catalpifolium to Acid Stress
Source: Plants (Basel). 2021 Sep 19;10(9):1958. doi: 10.3390/plants10091958 (PMC8470873; doi:10.3390/plants10091958)
Supplement: Supplementary file 1 [file plants-10-01958-s001.zip › plants-1359830-supplementary.pdf]

Supportive information

Morphological, physiological and photophysiological responses of critically endangered *Acer catalpifolium* to acid stress

Yuyang Zhang [1,2], Tao Yu [2], Wenbao Ma[3,*], Buddhi Dayananda [4], Kenji Iwasaki[5] and Junqing Li[2,*]

1 The national-Local Joint Engineering Laboratory of High Efficiency and Superior-Quality Cultivation and Fruit Deep Processing Technology on Characteristic Fruit Trees, College of Plant Science, Tarim University, Alear 843300, China; muyu64@sina.com

2 Beijing Key Laboratory for Forest Resources and Ecosystem Processes, Beijing Forestry University, Beijing 100083, China; yutao123@bjfu.edu.cn

3 Ecological Restoration and Conservation of Forests and Wetlands Key Laboratory of Sichuan Province, Sichuan Academy of Forestry, Chengdu 610081, China

4 School of Agriculture and Food Sciences, the University of Queensland, Brisbane, QLD 4072, Australia; b.dayananda@uq.edu.au

5 Climate Change Cluster (C3), Faculty of Science, University of Technology Sydney, Sydney, NSW 2007, Australia; Kenji.Iwasaki@alumni.uts.edu.au

* Correspondence: mawenbao_2021@163.com (W.M.); lijq@bjfu.edu.cn (J.L.)

Table S1 Three-way analysis of variance for the effects of acid treatments on growth, leaf morphological, biomass, gas eachange and chlorophyll parameters of *A. catalpifolium*

Types	Parameters	AAM	AT	AA	AAM*AT	AAM*AA	AT*AA	AAM*AT*AA
Growth parameters	Plant height	243.10***	7.96**	0.08	2.30	117.55***	0.08	0.22
	Ground stem	883.62***	14.94***	17.27***	46.24***	6.43*	8.54**	0.54
	Crown	676.64***	71.49***	83.87***	21.28***	460.05***	0.11	1.54
Leaf morphological parameter	Leaf length	26.08***	16.19***	1.76	0.18	25.20***	5.00*	0.16
	Leaf width	0.03	0.54	0.20	0.54	4.23*	0.91	0.43
	Leaf area	5.82*	1.78	7.68**	0.59	6.76*	1.52	23.48***
	Leaf weight	0.03	0.54	0.20	0.54	4.23*	0.91	0.43
	SLA	9.48**	13.70***	12.95***	0.02	50.58***	0.03	36.42***
Biomass parameters	Root	78.57***	9.85**	0.28	3.23	456.65***	5.82*	39.52***
	Leaf	2.11	4.82*	2.03	225.00***	266.34***	19.15***	2.71
	Lateral branch	69.13***	16.50***	26.36***	121.05***	383.38***	0.872	0.04
	Stem	190.16***	1.69	68.43***	84.30***	586.64***	16.36***	12.60***
	Total biomass	170.85***	0.01	16.92***	102.95***	1344.66***	10.44**	44.68***
	RSR	0.24	44.81***	5.13*	274.28***	44.80***	39.82***	0.33
Gas exchange indices	Pn	986.84***	56.42***	28.64***	0.76	86.33***	12.23***	4.56*
	Cs	11.30**	555.34***	0.75	1.08	5.24*	34.73***	40.06***
	Ci	1568.78***	21.13***	61.15***	0.02	1.75	7.62**	17.95***
	Tr	1.63	189.25***	136.31***	35.89***	48.09***	0.91	81.40***
	Ls	1026.23***	102.10***	0.25	597.15***	102.63***	2.05	7.37**
	WUE	271.74***	54.15***	0.09	139.59***	196.74***	51.83***	47.14***
Chlorophyll parameters	Chla	0.01	1.6	0.59	6.60*	0.55	2.46	5.63*
	Chlb	2.32	0.46	0.20	0.01	2.65	11.65**	5.03*

Total Chl	0.29	0.46	0.45	2.84	1.15	5.07*	5.66*
Chl a/b	8.06**	28.36***	3.13	34.85***	0.77	5.96*	7.43**
Carotenoids	1.97	0.42	1.80	0.34	1.09	7.73**	7.34**

Note: Numbers in the table represent F value of three-way analysis of variance. AAM, AT and AA stand for acid application methods, acid types and acidity of acid treatments. . *, **, *** indicate significance at 0.05, 0.01, <0.001 level.

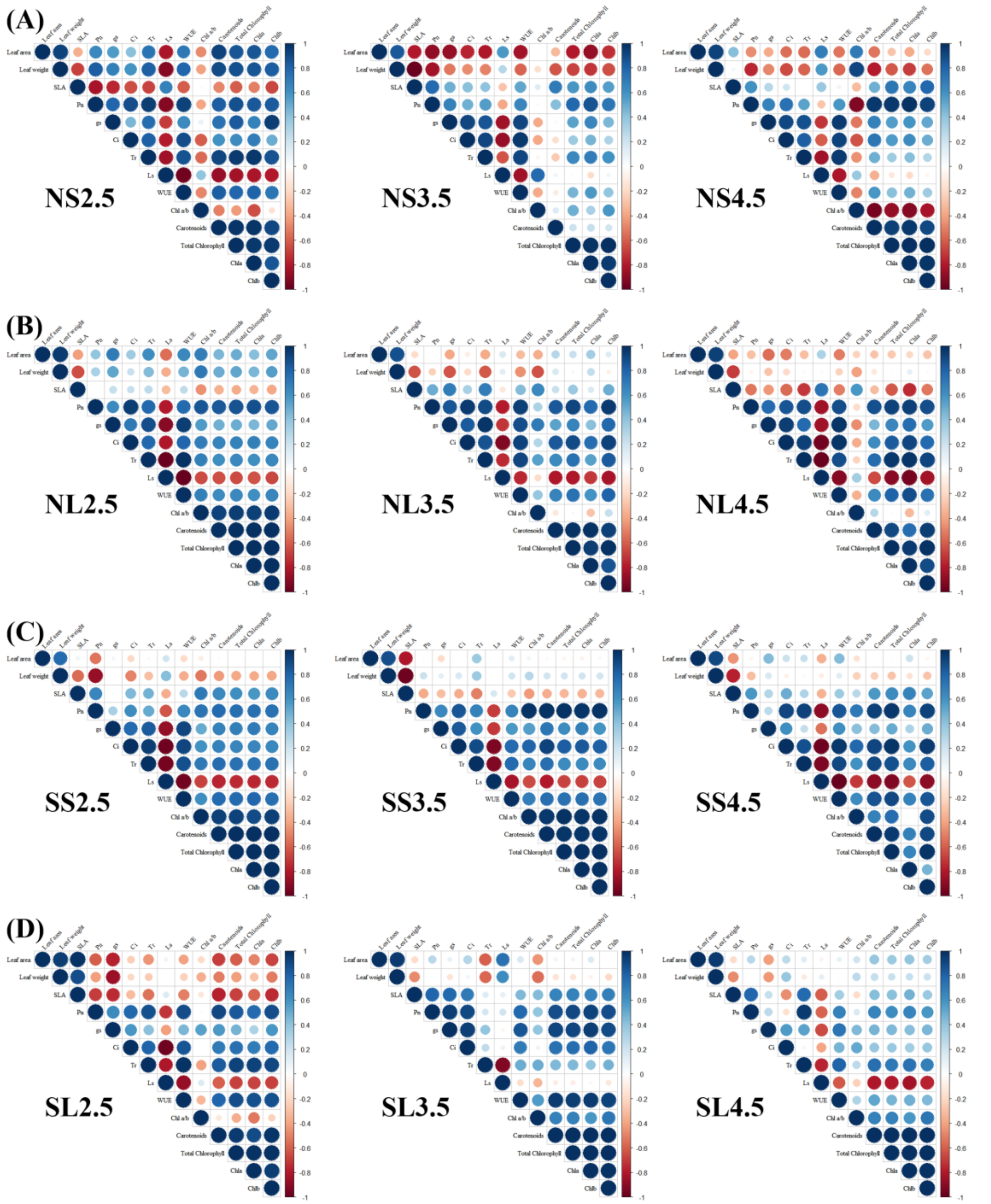

Figure S1. Correlation analysis of different foliar morphological and physiological indices of *Acer catalpifolium* under different acidic treatments.
